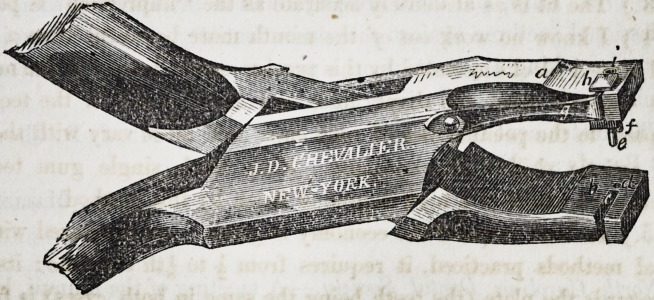# Improvement in Adjustable Punches for Setting Artificial Teeth

**Published:** 1857-01

**Authors:** Saml. Mallett


*Improvement in Adjustable Punches for Setting Artificial Teeth.*—This improvement consists in making an adjustable punch in such a manner, that the pins in the mineral teeth, will guide the punches so that they shall punch the holes in the plate to exactly correspond with the pins in the teeth. The punch is made in any of the ordinary forms of plyers or nippers, as shown in the drawing, or otherwise, having the levers or bars of suitable length, to use the punch with ease in punching the plate.

The improvement includes the combination of two punches, one immovable and the other movable in the slot, (*h*) with a spring (*g*)

and the two cavities, one (*i*) in the plug or immovable punch, and the other (*h*) movable with the movable punch, the latter to be set by the inserting of the wires of the teeth into the cavities, in order to make the distances between the holes in the plate correspond with the distance of the pins in the teeth as described.

To use this punch we place one of the pins of the tooth into the hole (*i*) and force out the movable punch far enough to allow the other pin in the tooth to pass into the slot, (*h*) which will adjust the two punches to exactly the distance of the pins, so that when the plate is punched the holes in the plate will receive the pins with perfect accuracy in every case.

This punch will be equally applicable for other analogous articles as well as teeth, where the pins may be at unequal distances in different cases.

The advantages of this punch over others are that it punches the holes for both pins at once, and that with great accuracy, if the pins in the teeth are straight, the punches being regulated in every case by the pins in the tooth. 2d, It may be used either for a double punch or a single one, by removing the movable punch, which can be done almost instantly, we can change it from a double to a single one, and do away with the necessity of having any other in the laboratory.

Also, there are two attachments to the instrument, and while they form no part of the punch proper they may be made very useful, and be used or not in connection with it. One is for bending the backing so that it will fit the convexity of the surface of the tooth when it is riveted, and the other is for countersinking the backings by pressure with a blunted point on the edges of the holes instead of reaming them out.

This improvement is by Samuel Mallett and Augustus B. Smith, of New Haven, Conn., and although the instrument was patented by them June 17th, 1856, and there are prejudices in the minds of some of our professional brethren against patents in dentistry, we can see no impropriety in taking a patent upon a purely mechanical instrument, and which is to be used exclusively in the laboratory.

This instrument is now being manufactured by Mr. J. D. Chevalier, 360 Broadway, New York.

Saml. Mallett.

## Figures and Tables

**Figure f1:**